# A Novel Spiral Si Drift Detector with a Constant Cathode Gap and Arbitrary Cathode Pitch Profiles

**DOI:** 10.3390/mi17030354

**Published:** 2026-03-13

**Authors:** Hongfei Wang, Zheng Li

**Affiliations:** 1 School of Physics and Physical Engineering, Qufu Normal University, Qufu 273165, China; wanghf.20@163.com; 2 School of Integrated Circuits, Ludong University, Yantai 264025, China; 3 Engineering Research Center of Photodetector Special Chip in Universities of Shandong, Ludong University, Yantai 264025, China

**Keywords:** spiral silicon drift detector, Arbitrary Pitch Profiles, constant cathode gap, electric field distribution, electric potential distributions, electron concentration distribution

## Abstract

In this paper, an innovative design of a silicon spiral drift detector (SDD) has been proposed. In this design, gaps under the SiO_2_ layer between the cathode rings are kept constant, with a minimum value to reduce the surface leakage current. The cathode pitch profile $P\left(r\right)$ as a function of radius r is allowed to change in an arbitrary way to achieve the optimum field distribution. The concept, design considerations, modeling and electrical simulations have been carried out for this novel structure with a hexagonal spiral silicon drift detector. Using one-dimensional analyses, we obtain the exact solution of the spiral design $r=r\left(\theta \right)\;$ with a near-arbitrary pitch profile $P\left(r\right)=P_{1}{\left(\frac{r}{r_{1}}\right)}^{\frac{1}{\eta }}$, with η as an arbitrary real number. We also obtained the electric potential and field profiles on both surfaces of the detector. Using a Technology Computer-Aided Design (TCAD) tool, we made 3D simulations of the detector’s electrical properties. The hexagonal spiral silicon drift detector has excellent electrical properties: a uniform electric field, smooth distribution of electric potential and electron concentration, and a clear electron drift channel. The distributions of the electric field, electric potential, and electron concentration are symmetrical and smooth, which is beneficial for applications in photon sciences (X-ray) and safeguards and homeland security (particle radiation). The theoretical work and simulation results serve as solid foundations for the detector design and the expansion of semiconductor technology.

## 1. Introduction

The concept of the silicon drift detector (SDD) was first introduced by E. Gatti and P. Rehak in the 1980s [1], marking a significant advancement in radiation detection technology. Since their emergence in 1983, SDDs have evolved as a pivotal tool across numerous scientific and industrial domains due to their exceptional performance and technological maturity. These detectors are fabricated using planar processing techniques and the principle of lateral depletion on ultra-pure, high-resistivity silicon substrates. In contrast to conventional silicon-based detectors like micro-strip or pixel devices, SDDs offer distinct benefits including rapid response times, high energy resolution, ease of integration, low capacitance, minimal noise, and uniform internal drift characteristics. Moreover, they operate effectively without the need for liquid nitrogen cooling, and under a nearly parallel surface drift field, the electrons move toward the anode at an approximately constant velocity.

SDDs have found extensive applications in cutting-edge fields such as in experiments at CERN’s LEP NA45 in 1992 and BNL’s Relativistic Heavy Ion Collider [2,3,4] in 2001, as well as in photon and nuclear physics [5,6], dark matter detection [7], X-ray fluorescence spectroscopy [8,9,10], medical imaging [11,12,13,14,15], and X-ray pulsar navigation [15,16,17,18,19]. For instance, pulsar-based navigation techniques leverage SDDs for deep space exploration and autonomous satellite guidance, with collaborative efforts like those between NASA and BNL focusing on modular X-ray spectrometers for lunar surface mapping. The historical progression of detector technology, beginning with single crystals, bipolar transistors, germanium crystal counters, lithium-drift detectors, and charge-coupled devices in the 1950s, culminated in the development of SDDs, underscoring their transformative impact.

Structurally, SDDs have advanced from initial silicon strip designs to concentric ring and spiral configurations. Traditional concentric ring SDDs feature a central anode surrounded by cathode rings but require manual voltage biasing for each ring to establish a potential gradient, leading to challenges such as nonlinear potential distribution and complex resistor selection. In response, the spiral SDD was developed to address the limitation of automatic voltage division. By utilizing a spiral cathode ring that functions simultaneously as a rectifying junction and voltage divider, this design generates a potential gradient (or transverse drift field), enabling incident carriers to drift efficiently to the collection anode [20,21] without external dividers. This innovation significantly simplifies operation and mitigates issues like high power consumption and heating, which are prevalent in large-area SDDs or arrays. The main advantages of the spiral SDDs are less surface leakage current (less noise, better resolution) and better drift field for fast charge collection. Consequently, the spiral SDD has become a mainstream technology.

In our novel design method, the cathode gaps (g) under SiO_2_ are kept constant to minimize surface leakage current. We allow the pitch profile $P\left(r\right)\;$ to vary in an arbitrary way to obtain the optimum electric field distribution. Our design is different from that in [22], where a constant electric surface field is imposed. Although cathode gaps were kept constant in both cases, our electric field is allowed to change to obtain the optimal profile. Exact solutions of SDD of spiral design $r=r\left(\theta \right)$ can be obtained for the hexagonal spiral SDD. Based on the theoretically calculated coordinates of each point on the spiral ring, the SDD structure diagram can be drawn. Technology Computer-Aided Design (TCAD) is used for simulation in this work. We have systematically researched its respective electrical properties, such as the electric field and potential distributions, and the electron concentration distribution.

## 2. The Novel Spiral Silicon Drift Detector (SDD) Concept and Design Considerations

**Figure 1:**
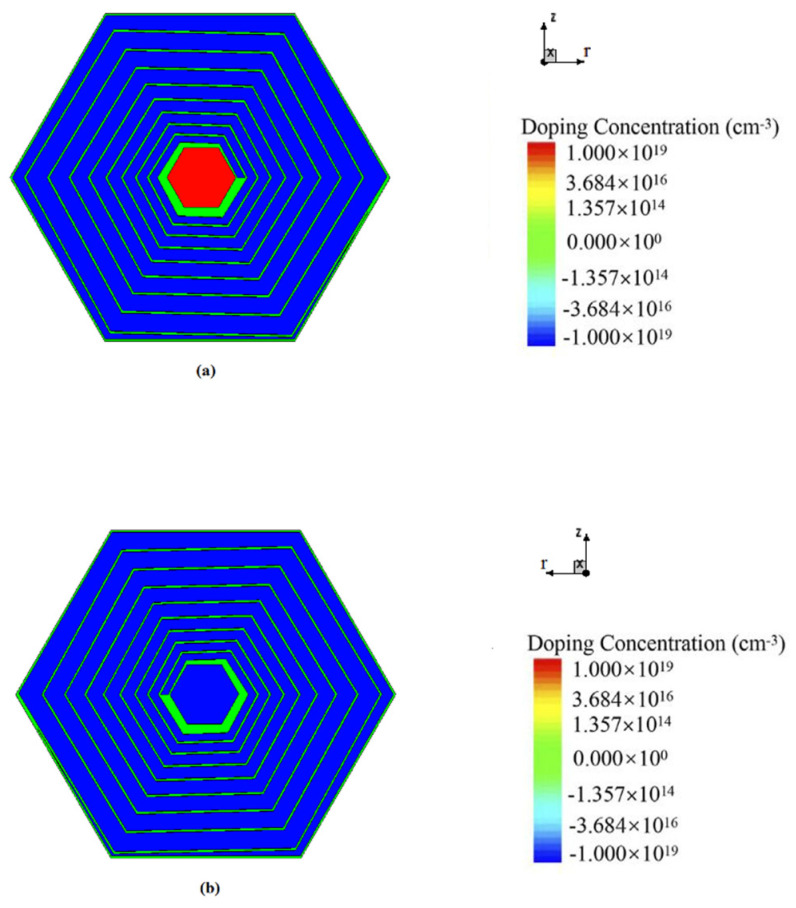
(**a**) The top view of the novel spiral SDD unit cell. (**b**) The bottom view of the unit cell.

**Figure 2:**
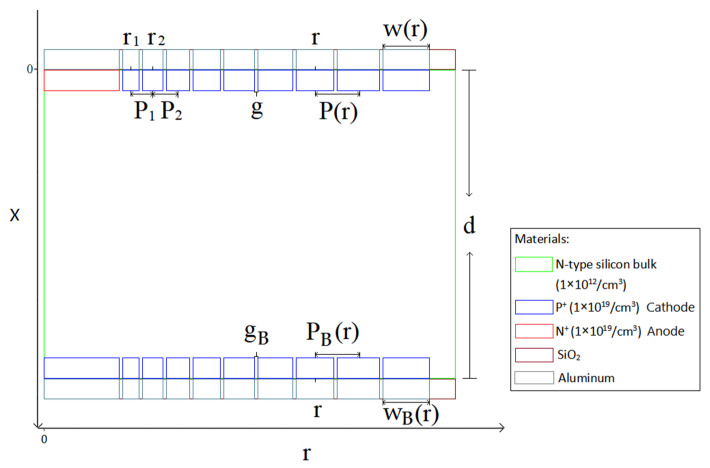
The cross-section view and the structural parameters of the novel spiral SDD.

Figure 1 presents the top and bottom views of the novel spiral SDD. The structural parameters and the foundational rules of the detector’s design can be seen clearly in Figure 2. In a spiral SDD, the cathode pitch $P\left(r\right)$, the cathode width $w\left(r\right)$, and the gap between cathode rings $g\left(r\right)$ can be written as, on the top surface:

(1) $$P\left(r\right)=g\left(r\right)+w\left(r\right)=g+w\left(r\right)$$

Here, we use constant gap g, and on the bottom surface

(2) $$P_{B}\left(r\right)=g_{B}(r)+w_{B}(r)=g_{B}+w_{B}(r)$$

In our work, we chose the constant and minimum gap values for both surfaces to reduce surface leakage current. On the top surface, let $E\left(r\right)$ as the electric field profile on the surface; then, the voltage drop between r and r+∆r is $\Delta V\left(r\right)$:

(3) $$\Delta V\left(r\right)=E\left(r\right)\cdot P\left(r\right)=I\frac{\rho_{s}}{w\left(r\right)}\cdot \alpha r$$

where $\rho_{s}$ denotes the sheet resistance of the cathode ion implant, and I represents the ohmic current in the spiral, and

$$\alpha =\left\{\begin{array}{c} 2\pi \;\left(circle\right)\\ 6\;(\mathrm{hexagon}) \end{array}\right.$$

where α is a parameter for different spiral shapes.

We have:

(4) $$E\left(r\right)=\frac{\alpha I\rho_{s}r}{P\left(r\right)w\left(r\right)}$$

From Equations (1) and (4), we have:

(5) $$E\left(r\right)=\frac{\alpha I\rho_{s}r}{P\left(r\right)[P\left(r\right)-g]}$$

We can obtain the electric potential profile $\phi \left(r\right)$ on the top surface:

(6) $$\phi \left(r\right)=\int_{r_{1}}^{r}E\left(r\right)dr=\alpha I\rho_{s}\int_{r_{1}}^{r}\frac{rdr}{P\left(r\right)[P\left(r\right)-g]}$$

We chose the following cathode pitch profile:

(7) $${P\left(r\right)=P_{1}\left(\frac{r}{r_{1}}\right)}^{\frac{1}{\eta }}$$

where a real number (near arbitrary) (0<η<∞), and η defines how fast $P\left(r\right)$ changes with r. Let

(8) $$x={\left(\frac{r}{r_{1}}\right)}^{\frac{1}{\eta }}$$

We have:

$$P\left(x\right)=P_{1}x$$

and

$$r=r_{1}x^{\eta }$$

where $r_{1}$ denotes the radius of the first ring of the spiral, and $P_{1}$ represents the pitch of the first ring.

Equation (6) becomes:

$$\phi \left(r\right)=\int_{1}^{x}\rho_{s}\alpha Ir_{1}\eta \frac{x^{\eta -1}dxr_{1}x^{\eta }}{P_{1}x\left(P_{1}x-g\right)}$$

or

(9) $$\phi \left(r\right)=\frac{\rho_{s}\alpha I{r_{1}}^{2}\eta }{P_{1}}\int_{1}^{x}\frac{x^{2\eta -2}dx}{P_{1}x-g}$$

If η=1,

(10) $$\phi \left(r\right)=\frac{2\alpha \rho_{s}I{r_{1}}^{2}\eta }{{P_{1}}^{2}}\ln\frac{{\left(\frac{r}{r_{1}}\right)}^{\frac{1}{2}}-\frac{g}{P_{1}}}{1-\frac{g}{P_{1}}}$$

(η=1)

For η≠1.

For

(11) $$g<\frac{P_{1}}{2}$$

Let

(12) $$y={\left(\frac{r}{r_{1}}\right)}^{\frac{1}{\eta }}-\frac{g}{P_{1}}>\frac{g}{P_{1}}(since{r\geq r}_{1},{\left(\frac{r}{r_{1}}\right)}^{\frac{1}{\eta }}\geq 1)$$

i.e.,

$$r>r_{1}{\left(2\frac{g}{P_{1}}\right)}^{\eta }(0<\eta <\infty )always\;stands$$

Since $y=x-\frac{g}{P_{1}}$,$\;x=y+\frac{g}{P_{1}}$,dx=dy

Equation (9) becomes

$$\phi \left(r\right)=\frac{\alpha \rho_{s}I{r_{1}}^{2}\eta }{{P_{1}}^{2}}\int_{1}^{x}\frac{x^{2\eta -2}dx}{x-\frac{g}{P_{1}}}=\frac{\alpha \rho_{s}I{r_{1}}^{2}}{{P_{1}}^{2}}\int_{y_{1}}^{y}\frac{{\left(y+\frac{g}{P_{1}}\right)}^{2\eta -2}}{y}dy$$

(13) $$\phi \left(r\right)=\frac{\alpha \rho_{s}I{r_{1}}^{2}\eta }{{P_{1}}^{2}}\int_{y_{1}}^{y}y^{2\eta -3}{\left(1+\frac{g}{P_{1}y}\right)}^{2\eta -2}dy$$

From Equation (12), we have

(14) $$\frac{g}{P_{1}y}<1$$

So, we can use the Taylor expansion to express:

(15) $$\begin{array}{c} {\left(1+\frac{g}{P_{1}y}\right)}^{2\eta -2}=1+\frac{\left(2\eta -2\right)}{1!}\left(\frac{g}{P_{1}y}\right)+\\ \;\;\;\;\;\;\;\;\;\;\;\;\;\;\;\;\;\;\;\;\;\;\;\;\;\;\;\;\;\;\;\;\;\;\;\;\;\;\;\;\;\;+\frac{\left(2\eta -2\right)\left(2\eta -3\right)}{2!}{\left(\frac{g}{P_{1}y}\right)}^{2}+\ldots \\ +\frac{\left(2\eta -2\right)\left(2\eta -3\right)\left(2\eta -4\right)\ldots \left(2\eta -i-1\right)}{i!}{\left(\frac{g}{P_{1}y}\right)}^{i}+\ldots \\ \;\;\;\;\;\;\;\;\;\;\;\;\;\;\;\;\;\;\;\;\;\;\;\;\;\;\;\;\;\;\;\;\;\;\;\;\;\;\;\;\;\;\;\;\;\;\;\;\;\;\;\;\;\;\;\;\;\;\;\;\;\;\;\;\;\;\;\;\;\;\;\;\;\;\;\;\;\;\;\;\;\;\;\;\;\;\;\;\;\;\;\;\;\;\;\;\;\;\;\;\;\;\;\;\;\;\;\;\;\;\;\;\;\;\;\;\;\;\;\;\;\;\;\;\;\;\;\;\;\;\;\left(i\to \infty \right)\;\; \end{array}$$

Equation (13) becomes

(16) $$\begin{array}{c} \;\phi \left(r\right)=\frac{\alpha \rho_{s}I{r_{1}}^{2}\eta }{{P_{1}}^{2}}\int_{y_{1}}^{y}y^{2\eta -3}dy\left[1+\right.\;\sum_{i=1}^{\infty }\frac{\left(2\eta -2\right)\left(2\eta -3\right)\ldots \left(2\eta -i-1\right)}{i!}{\left(\frac{g}{P_{1}y}\right)}^{i}]\; \end{array}$$

Carry out integration, we have

(17) $$\begin{array}{c} \phi \left(r\right)=\frac{\alpha \rho_{s}I{r_{1}}^{2}\eta }{{P_{1}}^{2}}[\frac{\left(y^{2\eta -2}-y_{1}^{2\eta -2}\right)}{2\eta -2}+\\ +\sum_{i=1}^{\infty }\frac{\left(2\eta -2\right)\left(2\eta -3\right)\ldots \left(2\eta -i-1\right)}{\left(2\eta -2-i\right)i!}(y^{2\eta -2-i}-y_{1}^{2\eta -2-i})]+V_{E1} \end{array}$$

where

(18) $$\left\{\begin{array}{c} y={\left(\frac{r}{r_{1}}\right)}^{\frac{1}{\eta }}-\frac{g}{P_{1}}\\ y_{1}=1-\frac{g}{P_{1}} \end{array}\right.$$

We can use the boundary conditions (we already used one of them in Equation (17)):

(19) $$\left\{\begin{array}{c} \phi \left(r=r_{1}\right)=V_{E_{1}}\\ \phi \left(r=R\right)=V_{out} \end{array}\right.$$

Let

$$\begin{array}{c} A=\frac{\alpha \rho_{s}I{r_{1}}^{2}\eta }{{P_{1}}^{2}}\;\; \end{array}$$

and

$$\begin{array}{c} B\left(\frac{r}{r_{1}}\right)=\frac{\left(y^{2\eta -2}-y_{1}^{2\eta -2}\right)}{2\eta -2}+\\ +\sum_{i=1}^{\infty }\frac{\left(2\eta -2\right)\left(2\eta -3\right)\cdots \left(2\eta -i-1\right)}{\left(2\eta -2-i\right)i!}(y^{2\eta -2-i}-y_{1}^{2\eta -2-i}) \end{array}$$

We can rewrite Equations (17) and (19) as:

(20) $$\left\{\begin{array}{c} \phi \left(r\right)=A\cdot B\left(\frac{r}{r_{1}}\right)+V_{E1}\\ \phi \left(r=r_{1}\right)=A\cdot B\left(1\right)+V_{E1}=V_{E1}\\ \phi \left(r=R\right)=A\cdot B\left(\frac{R}{r_{1}}\right)+V_{E1}=V_{out} \end{array}\right.$$

We have:

$$V_{out}-V_{E1}=A\cdot B\left(\frac{R}{r_{1}}\right)$$

Finally, we have the design value of $P_{1}$:

(21) $$P_{1}=\sqrt{\frac{\alpha \rho_{s}I{r_{1}}^{2}\eta }{V_{out}-V_{E1}}\cdot B\left(\frac{R}{r_{1}}\right)}$$

For the backside, we let the backside surface electric potential

(22) $$\Psi_{B}\left(r\right)=V_{B}+\gamma \phi \left(r\right)\;\;\;\left(\gamma <1\right)$$

$V_{B}$ is the voltage on the central cathode on the backside. We can calculate the circular SDD spiral rings’ polar angle $\theta \left(r\right)$ (top surface) [19]:

(23) $$\theta \left(r\right)=\int_{r_{1}}^{r}\frac{2\pi dr}{P\left(r\right)}$$

Using Equation (7), we have:

(24) $$\theta \left(r\right)=\frac{2\pi r_{1}}{P_{1}}\frac{1}{1-\frac{1}{\eta }}\left.[{\left(\frac{r}{r_{1}}\right)}^{1-\frac{1}{\eta }}-1\right.]$$

Then, for SDD spiral rings we have

(25) $$\theta \left(r\right)=\left\{\begin{array}{c} 2\pi \frac{r_{1}}{P_{1}}\ln\frac{r}{r_{1}}\;\;\;\;\;\;\;\;\;\;\;\;\;\;\;\;\;(\;\eta =1)\;\\ \;2\pi \frac{r_{1}}{P_{1}}\frac{1}{1-\frac{1}{\eta }}\left[{\left(\frac{r}{r_{1}}\right)}^{1-\frac{1}{\eta }}-1\right]\;\;\;\;\;\;\;\;\;(\eta >1)\;\; \end{array}\right.$$

Solve r as the function of θ,

(26) $$r=\left\{\begin{array}{c} r_{1}e^{\frac{P_{1}}{r_{1}}\frac{\theta }{2\pi }}\;\;\;\;\;\;\;\;\;\;\;\;\;\;\;\;\;\;\;\;(\;\eta =1)\;\\ r_{1}{\left[1+\frac{P_{1}}{2\pi r_{1}}\frac{\eta -1}{\eta }\theta \right]}^{\frac{\eta }{\eta -1}}\;\;\;\;\;\;\;\;\;\;\;\;(\eta >1) \end{array}\right.$$

For the backside, from Equation (22), we have the backside electric field:

(27) $$E_{B}\left(r\right)=\gamma E\left(r\right)$$

Let

(28) $$P_{B}\left(r\right)=P\left(r\right)$$

then

(29) $$\theta_{B}\left(r\right)=\int_{r_{1B}}^{r}\frac{2\pi dr}{P_{B}\left(r\right)}=\int_{r_{1B}}^{r}\frac{2\pi dr}{P\left(r\right)}$$

Similarly to Equation (2), we have:

(30) $$\theta_{B}\left(r\right)=\frac{2\pi r_{1B}}{P_{1}}\frac{1}{1-\frac{1}{\eta }}\cdot \left[r^{1-\frac{1}{\eta }}-{r_{1B}}^{1-\frac{1}{\eta }}\right]$$

Let

(31) $$r_{1B}=r_{1}$$

We have

(32) $$\theta_{B}\left(r\right)=\theta \left(r\right)$$

(33) $$r_{B}=\left\{\begin{array}{c} r_{1}e^{\frac{P_{1}}{r_{1}}\frac{\varphi }{2\pi }}\;\\ r_{1}{\left[1+\frac{P_{1}}{2\pi r_{1}}\frac{\eta -1}{\eta }\varphi \right]}^{\frac{\eta }{\eta -1}} \end{array}\right.$$

Since:

(34) $$P_{B}\left(r\right)=g_{B}+w_{B}(r)$$

Let $g_{B}=g$, $w_{B}=N$, we get:

(35) $$P_{B}\left(r\right)=g+w_{B}\left(r\right)=g+w\left(r\right)=P\left(r\right)$$

and

(36) $$\left\{\begin{array}{c} \;P_{B}\left(r\right)=P_{1}{\left(\frac{r}{r_{1}}\right)}^{\frac{1}{\eta }}\;\;\\ w_{B}\left(r\right)=P_{1}{\left(\frac{r}{r_{1}}\right)}^{\frac{1}{\eta }}-g \end{array}\right.$$

Now, back to Equations (17) and (18). Let $\frac{g}{P_{1}}=0.1$ $\left(g=10\;\mu m,P_{1}=100\;\mu m\right)$, η=1.6, we have:

(37) $$\left\{\begin{array}{c} \;y\left(r\right)={\left(\frac{r}{r_{1}}\right)}^{\frac{1}{1.6}}-0.1\;\;\\ y_{1}=1-0.1=0.9 \end{array}\right.$$

Starting from the fourth term, the subsequent contributions are negligible due to their diminishing magnitude; thus, the truncated formula retains only the first three terms. Equation (17) is now:

(38) $$\begin{array}{r} \phi \left(r\right)=V_{E1}+\frac{1.6\rho_{s}I{r_{1}}^{2}}{P_{1}}\frac{y^{1.2}-y_{1}^{1.2}}{1.2}+\frac{1.6\rho_{s}I{r_{1}}^{2}}{P_{1}}\cdot 0.6\left(y^{0.2}-y_{1}^{0.2}\right)\\ +\;\;\;+\frac{1.6\rho_{s}I{r_{1}}^{2}}{P_{1}}\cdot \left[-0.015\left(y^{-0.8}-y_{1}^{-0.8}\right)\right] \end{array}$$

Here, we only take i=3 to achieve enough accuracy.

On the backside, we have:

(39) $$\Psi \left(r\right)=V_{B}+0.3\phi \left(r\right)$$

The detector full-depletion voltage is $V_{fd}$:

(40) $$V_{fd}=\frac{eN_{eff}d^{2}}{2\varepsilon \varepsilon_{0}}$$

Let $V_{B}=V_{fd}$, d=0.03 cm, we have $V_{fd}=72.09\;V$.

For R=1200 μm=0.12 cm, α=6, $\rho_{s}=2000,$ $I=10\times 10^{-5}\;A,\eta =1.6,$ g=0.001 cm, $r_{1}=0.0255\;cm,$ $V_{out}=30\;V,$ and $V_{E1}=18\;V,$ we have:

$$\left\{\begin{array}{c} y\left(R\right)={\left(\frac{R}{r_{1}}\right)}^{\frac{1}{1.6}}-0.1=2.633\\ y_{1}=1-0.1=0.9 \end{array}\right.$$

$$B\left(\frac{R}{r_{1}}\right)=\frac{y^{1.2}-y_{1}^{1.2}}{1.2}+0.6\left(y^{0.2}-y_{1}^{0.2}\right)-0.015\left(y^{-0.8}-y_{1}^{-0.8}\right)=2.0787$$

From Equation (21) we obtain:

$$P_{1}=0.014706\;cm\approx 147.1\;\mu m$$

**Figure 3:**
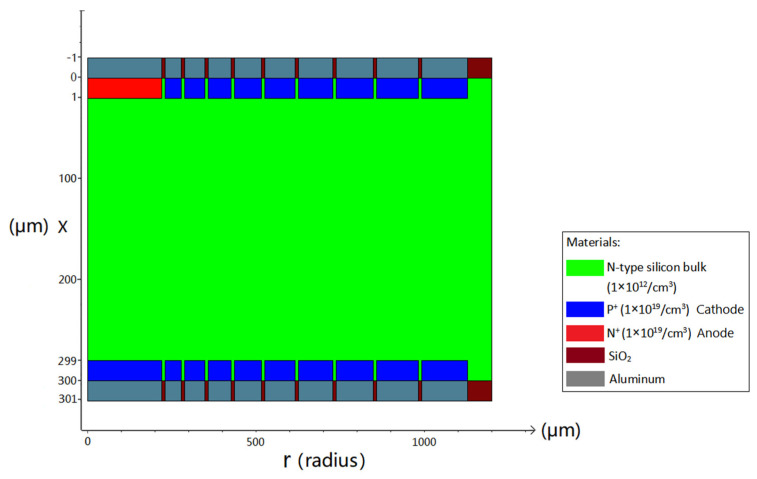
A two-dimensional cross-section view of the novel spiral SDD.

Due to the fact that the hexagonal shape is the closest to the circular shape and can also be arranged in an array to make a pixel detector, we select the hexagonal shape for the detector unit. An n-type silicon substrate with a doping level of 1 × 10^12^ cm^−3^ is used. The spiral SDD cell has a thickness of 300 μm. Figure 3 is a 2D cross-sectional view at Z=0 μm (due to symmetry, only half of the cross-section is shown). The structure of the spiral SDD anode (near r=0) and the cathode rings on both sides can be shown clearly. The aluminum electrode contact layers and the silicon dioxide protective layers are also observable. We can see that the central feature on the cell’s top surface is a heavily doped N-type region, which serves as the collecting anode and has a radius of 220.4 μm. The doped layer exhibits a concentration of 1 × 10^19^/cm^3^ and the doping depth is 1 μm. Heavily doped P-type spiral rings, with a doping depth of 1 μm and a doping concentration of 1 × 10^19^/cm^3^, are around the anode. The detector anode surface is covered with a 1 μm aluminum layer. Likewise, the start and end points of the spiral ring are also aluminized to the same thickness. The remaining surfaces are coated with a 1 μm silicon dioxide layer, utilizing the material’s inherent stability and chemical inertness. At the bottom of the silicon bulk, there is a heavily doped hexagonal P-type cathode with a radius of 220.4 μm near r=0. The doping concentration is 1 × 10^19^/cm^3^ and the doping depth is 1 μm. A heavily doped P-type spiral ring with a doping depth of 1 μm and a doping concentration of 1 × 10^19^/cm^3^ is surrounding the central cathode. The spiral ring on the top surface, along with the central hexagonal cathode on the bottom surface and the spiral ring jointly constitutes the cathode of this detector. The electrode contact points of the structure are positioned at the central positions of the anode top surface, the central cathode (bottom surface), the starting point of the first ring of the spiral cathode, and the ending point of the spiral cathode on both surfaces, respectively.

## 3. Electrical Characteristic Results

To investigate the electrical behavior performance of the spiral SDD in more detail, we carried out 3D simulations of detector electrical properties including the profiles of electric field, potential, and electron concentration by TCAD (Sentaurus 2019).

### 3.1. Electric Field Distribution of the Spiral SDD

**Figure 4:**
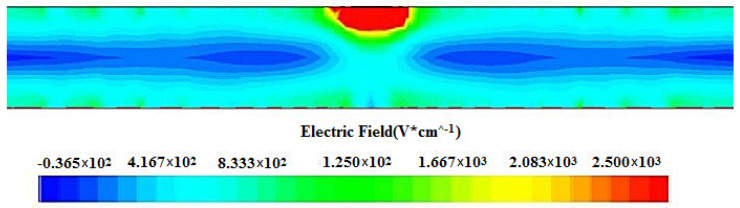
Electric field distribution of a cross-section at Z=0 μm through the detector cell.

To reveal the internal electric field distribution more clearly, we created a 2D cross-section at Z=0 μm through the detector cell shown in Figure 4. The red zones indicate strong electric field regions corresponding to the anode positions, while the central regions exhibit nearly stable electric field intensity with distinct drift channels, indicating the complete depletion of the detector. We can obviously see a nearly stable and uniform electric field distribution on the carrier drift channel, demonstrating a superior electric field uniformity that can enhance the detector performance.

**Figure 5:**
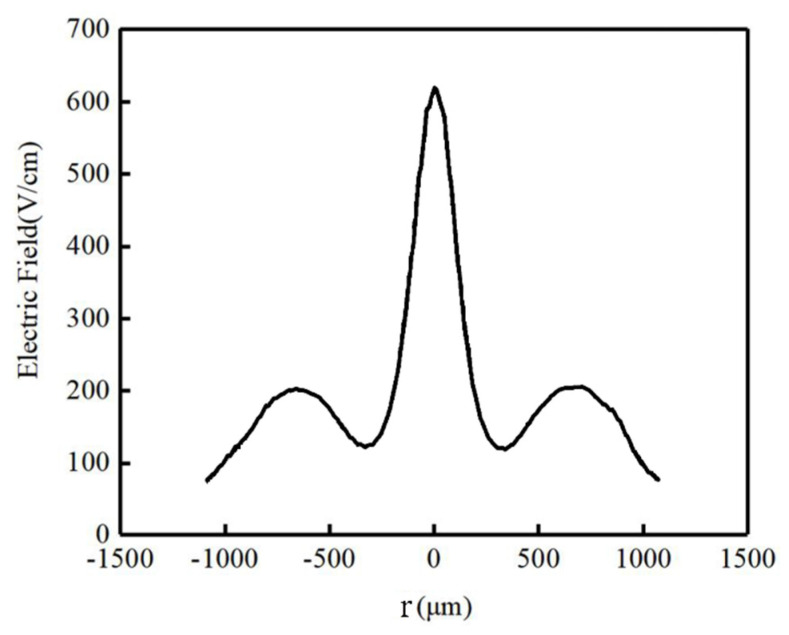
Electric field profile of the one-dimensional cross-section of the novel detector (X=160 μm).

Figure 5 presents a one-dimensional curve for a cut at the X=160 μm position from Figure 4. It is evident that the detector’s electric field intensity distribution exhibits an anode-symmetric pattern; the detector’s electric field is between 100 V/cm near the outer ring (r=R) and close to 600 V/cm near the anode.

### 3.2. Electric Potential Distribution of the Spiral SDD

**Figure 6:**
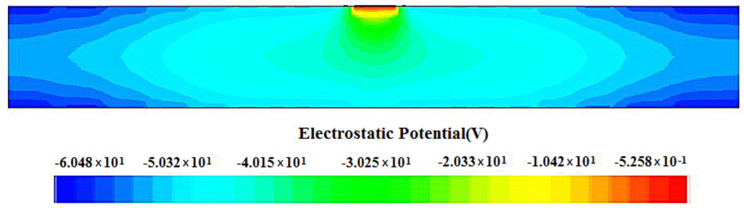
Electric potential profile of a cross-section at Z=0 μm through the detector cell.

An analysis of the electric potential distribution across a two-dimensional cross-section of the spiral SDD at Z=0 μm is shown in Figure 6. It is evident that the potential distribution is highly symmetrical. The figure shows that the potential is the highest in the collector anode region (red area), while the surrounding areas exhibit lower potential. Under the electric field force, carriers drift from low-potential regions to high-potential regions, moving from the periphery toward the detector center. They ultimately reach the collector anode to complete the charge collection. It demonstrates a good gradient distribution of potential.

**Figure 7:**
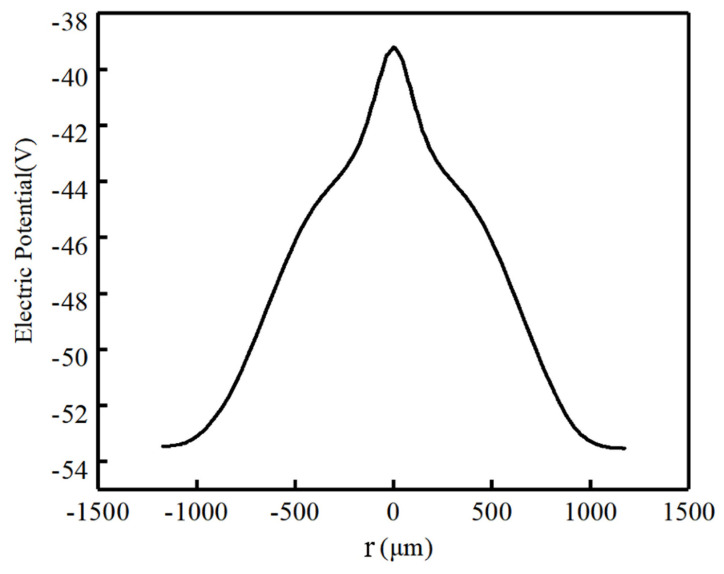
Electric potential profile of the one-dimensional cross-section of the novel detector (X=160 μm).

In Figure 7, we can see the one-dimensional potential distribution at a cut at X=160 μm from Figure 6. It is evident that the potential reaches its maximum at the anode and gradually decreases from the center toward both ends, exhibiting a smooth potential grading for electrons drifting towards the anode.

### 3.3. Electron Concentration Profile for the Spiral SDD

**Figure 8:**
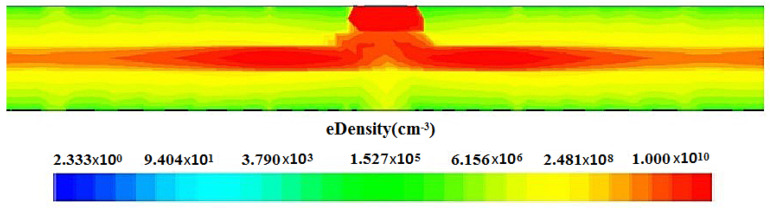
Electric concentration profile for the spiral SDD.

The 2D electron concentration distributions of the spiral SDD under full-depletion conditions are shown in Figure 8 (cut at Z=0). We can see that the electron concentration is the highest in the collector anode region, forming a clear drifting channel for the electrons (red area in the figure) in the detector’s central region, consistent with the drift channel demonstrated by the electric field distribution shown in Figure 4.

## 4. The Novel Spiral Silicon Drift Detector (SDD) Concept and Design Considerations from the Second Arbitrary P(r) Profile

A second arbitrary $P\left(r\right)$ profile:

Let

(41) $$P\left(r\right)=\frac{\xi P_{1}}{\xi +\sin\frac{r-r_{1}}{r_{1}}}\;\;(\xi \geq 2)$$

Then the spiral angle $\theta \left(r\right)$ is

$$\theta \left(r\right)=\int_{r_{1}}^{r}\frac{2\pi }{P\left(r\right)}dr=\frac{2\pi }{{\xi P}_{1}}\int_{r_{1}}^{r}\left[\xi +\sin\frac{r-r_{1}}{r_{1}}\right]dr$$

(42) $$\theta \left(r\right)=\frac{2\pi }{{\xi P}_{1}}\left[\xi \left(r-r_{1}\right)+r_{1}\left(1-\cos\frac{r-r_{1}}{r_{1}}\right)\right]$$

For a SDD front surface field $E\left(r\right)$,

since

(43) $$E\left(r\right)\cdot P\left(r\right)=\frac{\alpha I\rho_{s}r}{w\left(r\right)}$$

Let

(44) $$w\left(r\right)=\beta P\left(r\right)$$

Then we have

(45) $$E\left(r\right)=\frac{\alpha I\rho_{s}r}{\beta {P\left(r\right)}^{2}}$$

The SDD front surface potential is

(46) $$\phi \left(r\right)=\int_{r_{1}}^{r}E\left(r\right)dr+V_{E1}$$

From Equations (41) and (45) we get:

$$\phi \left(r\right)=\frac{\alpha I\rho_{s}}{\beta \xi P_{1}}\int_{r_{1}}^{r}{\left[\xi +\sin\frac{r-r_{1}}{r_{1}}\right]}^{2}dr+V_{E1}$$

(47) $$\phi \left(r\right)=\frac{\alpha I\rho_{s}}{\beta \xi P_{1}}\left[\left(\xi^{2}+\frac{1}{2}\right)\left(r-r_{1}\right)+2\xi r_{1}\cdot \left(1-\cos\frac{r-r_{1}}{r_{1}}\right)-\frac{r_{1}}{4}\sin\frac{2\left(r-r_{1}\right)}{r_{1}}+V_{E1}\right]$$

$$\phi \left(R\right)=V_{out}$$

We have:

(48) $$V_{out}-V_{E1}=\frac{\alpha I\rho_{s}}{\beta \xi P_{1}}\left[\left(\xi^{2}+\frac{1}{2}\right)\left(R-r_{1}\right)+2\xi r_{1}\cdot \left(1-\cos\frac{r-r_{1}}{r_{1}}\right)-\frac{r_{1}}{4}\sin\frac{2\left(r-r_{1}\right)}{r_{1}}\right]$$

This equation can be used to determine ξ or $P_{1}$ (if one or the other is fixed).

On the backside surface, we let:

(49) $$\left\{\begin{array}{c} P_{B}\left(r\right)=P\left(r\right)\\ w_{B}\left(r\right)=w\left(r\right)\\ \theta_{B}\left(r\right)=\theta \left(r\right)\\ \Psi \left(r\right)=V_{B}+\gamma \phi \left(r\right)\;\;\;\;\;\;\;\left(0<\gamma <1\right) \end{array}\right.$$

We can use the method of iteration to calculate r as a function of θ as the following:

(50) $$①\;\mathrm{let}\;r^{\left(0\right)}{=r}_{1}$$

(51) $$②\;r^{\left(1\right)}{=r}_{1}+\frac{\theta }{2\pi }P_{1}$$

(52) $$③\;r^{\left(2\right)}{=r}_{1}+\frac{1}{\xi }\left\{\frac{{\xi P}_{1}\theta }{2\pi }-r_{1}\left(1-\cos\frac{r^{\left(1\right)}-r_{1}}{r_{1}}\right)\right\}$$

(53) $$④\;r^{\left(n\right)}{=r}_{1}+\frac{1}{\xi }\left\{\frac{{\xi P}_{1}\theta }{2\pi }-r_{1}\left(1-\cos\frac{r^{\left(n-1\right)}-r_{1}}{r_{1}}\right)\right\}$$

(54) $$⑤\;\mathrm{stop}\;\mathrm{till}:\;\left|\frac{{r^{\left(n\right)}-r}^{\left(n-1\right)}}{r^{\left(n\right)}}\right|\leq 10^{-4}$$

We used the following parameters for SDD calculations:

$P_{1}=0.00638\;cm,r_{1}=0.0255\;cm,R=1200\;\mu m=0.12\;cm,$ ξ=2, β=0.85, we get a SDD with 21 turns of spiral.

**Figure 9:**
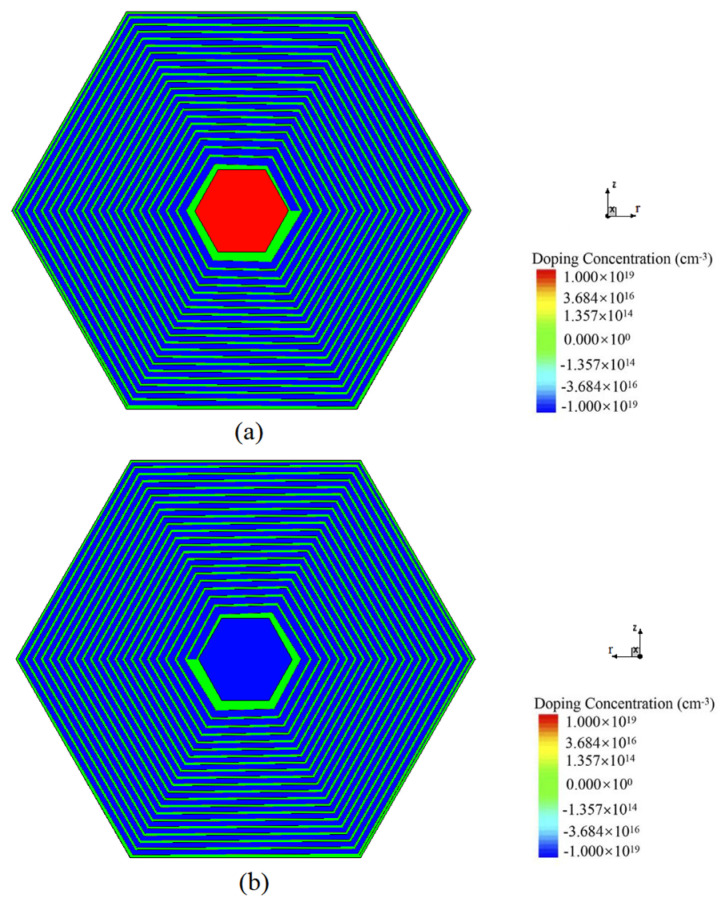
(**a**) The top view of the novel spiral SDD unit cell from the second arbitrary P(r) profile. (**b**) The bottom view of the unit cell from the second arbitrary P(r) profile.

**Figure 10:**
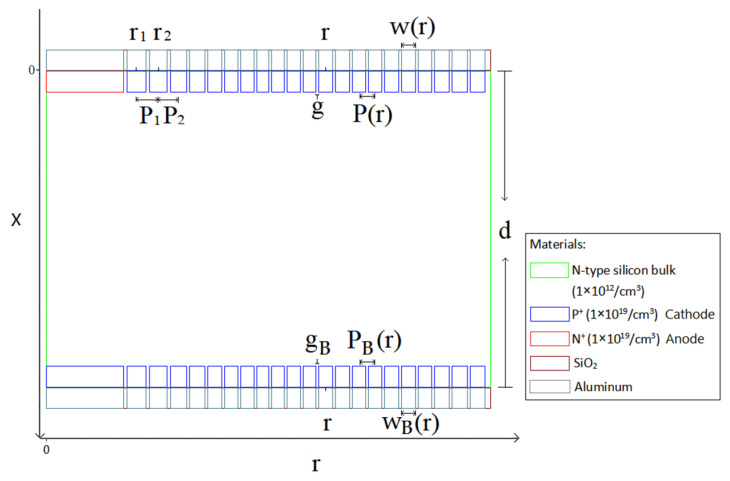
The cross-section view and structural parameters of the novel spiral SDD from the second arbitrary P(r) profile.

**Figure 11:**
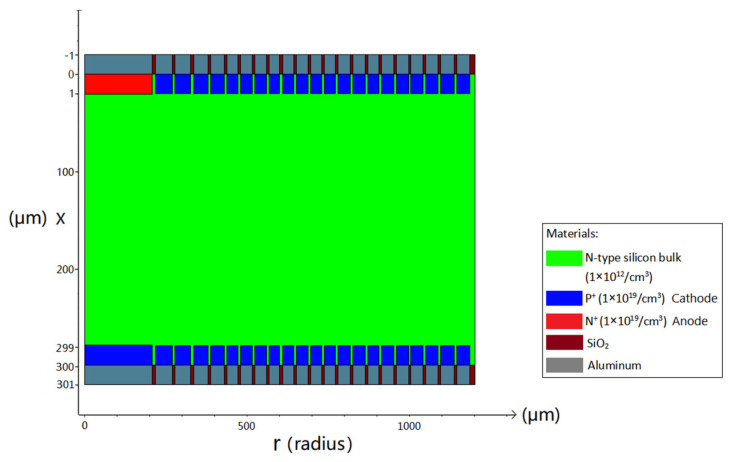
A two-dimensional cross section view of the novel spiral SDD from the second arbitrary P(r) profile.

Figure 9 presents the top and bottom views of the novel spiral SDD from the second arbitrary P(r) profile. The structural parameters and the foundational rules of the detector’s design from the second arbitrary P(r) profile can be seen clearly in Figure 10. Figure 11 is a 2D cross-sectional view at Z=0 μm from the second arbitrary P(r) profile (due to symmetry, only half of the cross-section is shown).

## 5. Electrical Characteristic Results from the Second Arbitrary P(r) Profile

### 5.1. Electric Field Distribution of the Spiral SDD from the Second Arbitrary P(r) Profile

**Figure 12:**
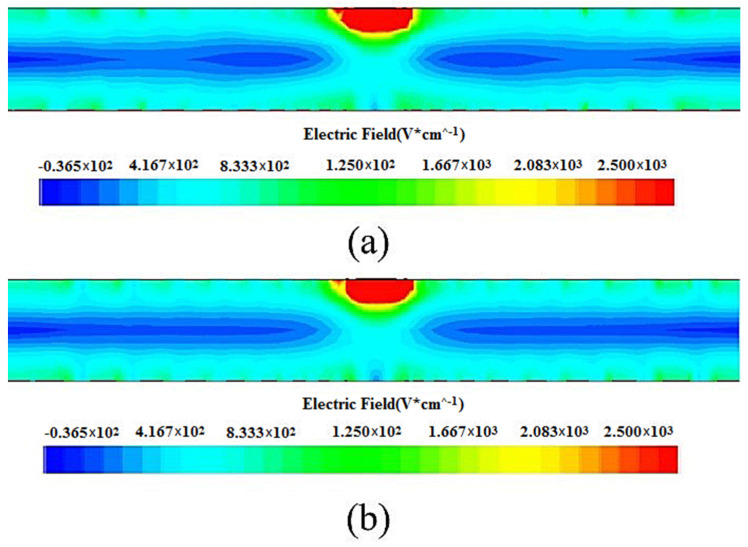
The electric field distribution diagrams of the spiral SDDs: (**a**) the P(r), (**b**) the second P(r).

Due to the appropriate pressure applied to the contact points of the electrodes of the double-sided spiral SDD, the detector has a reasonable potential distribution, and a good electric field distribution can be formed within the detector. Figure 12 shows the electric field distribution inside the P(r) and the second P(r) structures of the spiral SDDs, respectively. The red area is the strong electric field area where the detector anode is located. The electric field intensity in the middle area is almost stable and forms a distinct drift channel, indicating that the detector has been completely exhausted. It can clearly be seen from the figure that there is an uneven distribution of the electric field at the position of the carrier drift channel in the middle of the P(r) detector, while the electric field distribution in the middle area of the second P(r) detector is uniform and the carrier drift channel is nearly straight, indicating that the second P(r) detector has a higher uniformity of the electric field and better detector performance.

**Figure 13:**
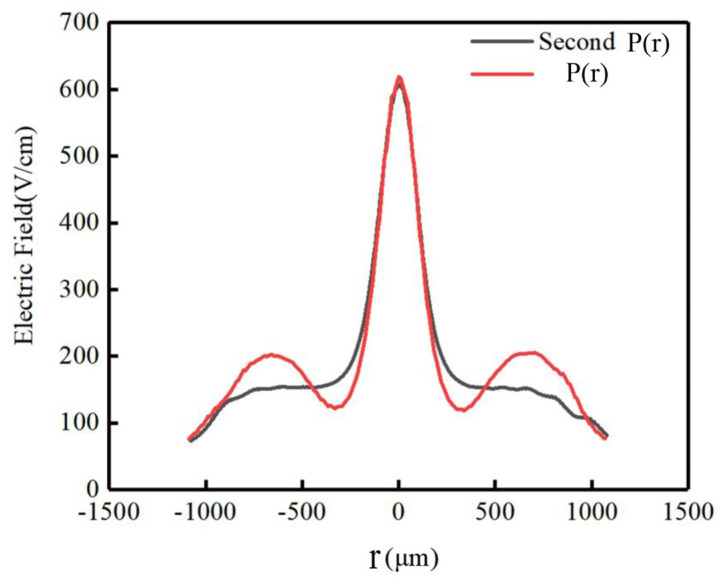
Electric field distribution curves of the spiral SDDs.

To compare the uniformity of the electric field distribution of the P(r) and the second P(r) detectors, Figure 13 shows a one-dimensional curve comparison of the electric field distribution at X=160 μm, with the red curve representing the electric field distribution of the P(r) detector and the black curve representing the electric field distribution of the second P(r) detector. It can be clearly observed that the electric field intensity distribution of the second P(r) detector is symmetrical about the anode, there is a peak in the electric field distribution curve, and the magnitude of the electric field decreases with the increase in detector radius, and the curve is smooth. The electric field intensity distribution of the P(r) detector has three peaks including the anode area, and the electric field variation trend is unstable with fluctuations. Thus, it is verified that the electric field distribution of the second P(r) detector is more uniform and has better electrical performance than that of the P(r) detector.

### 5.2. Electric Potential Distribution of the Spiral SDD from the Second Arbitrary P(r) Profile

**Figure 14:**
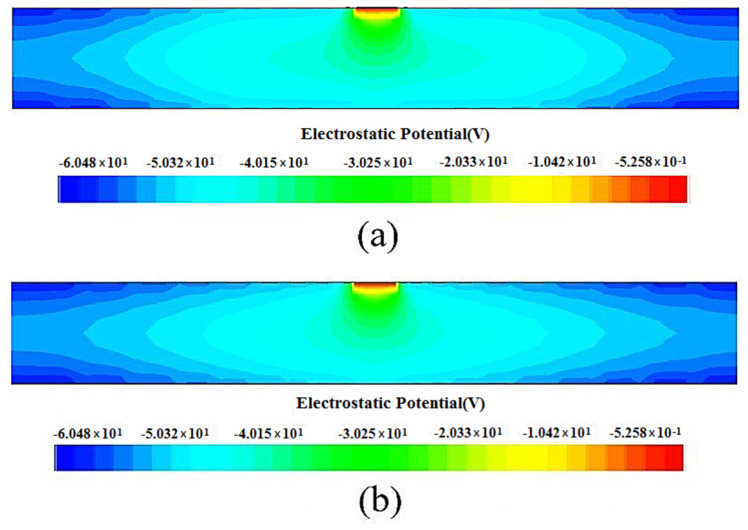
The two-dimensional cross-section potential distribution of the spiral SDDs: (**a**) the P(r), (**b**) the second P(r).

Figure 14 shows the two-dimensional cross-sectional potential distribution of the P(r) and the second P(r) structures of the double-sided spiral SDDs, respectively. It can be seen from the figure that the area where the collected anode is located has the highest potential (the red area in the figure), while the potential around it is low. The carriers drift under the force of the electric field, moving the path from the low-potential area to the high-potential area, that is, the carriers drift from the periphery to the center of the detector, and finally reach the collection anode to complete the signal readout. The second P(r) detector shown in Figure 14b has a more uniform, symmetrical and better gradient distribution of potential than the P(r) detector.

**Figure 15:**
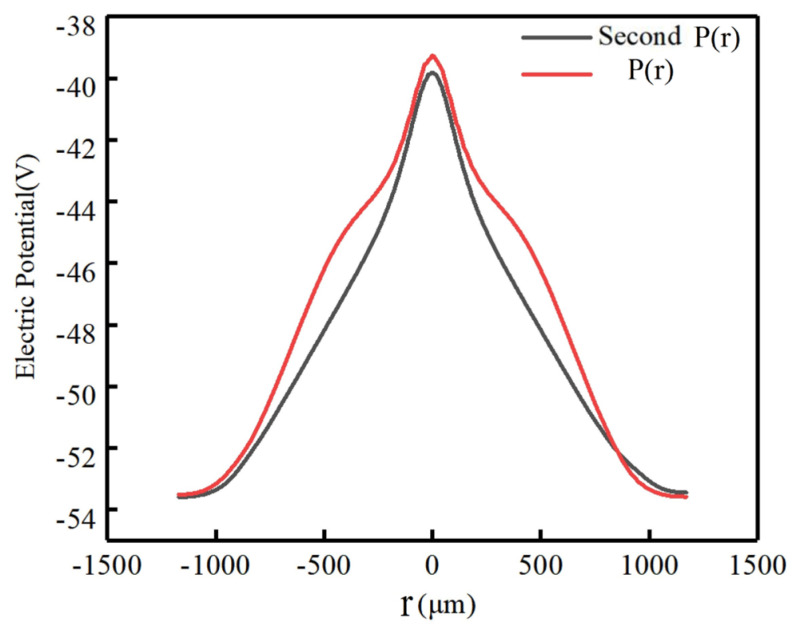
Potential distribution curves of the spiral SDDs.

To highlight the difference in potential distribution between the P(r) and the second P(r) detectors, the potential distribution of the detector was cut at X=160 μm to obtain the one-dimensional curve of potential with radius as shown in Figure 15. It can be clearly observed that the potential is highest at the anode and gradually decreases from the middle to both ends. The potential distribution curve of the second P(r) detector is smoother than that of the P(r) detector, meaning that the potential distribution of the second P(r) detector is more uniform.

### 5.3. Electron Concentration Profile for the Spiral SDD from the Second Arbitrary P(r) Profile

**Figure 16:**
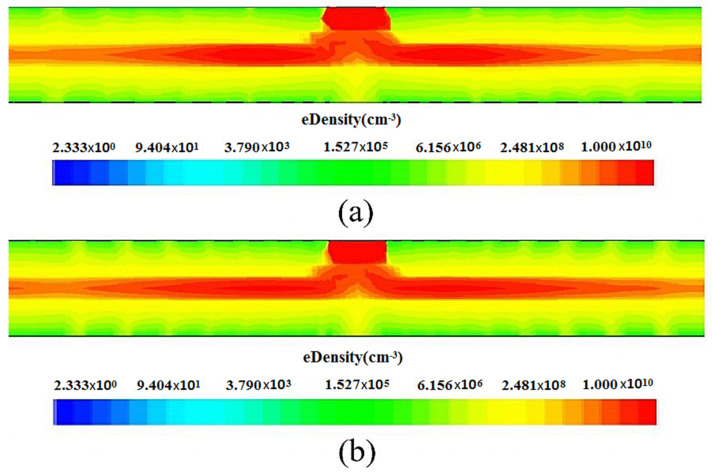
Electron concentration distribution diagrams of the spiral SDDs: (**a**) the P(r), (**b**) the second P(r).

Figure 16 respectively shows the electron concentration distribution of the P(r) and the second P(r) structures of the spiral SDDs in the fully exhausted state, with the highest electron concentration in the collected anode region and a high electron concentration channel (the red area in the figure) generated in the middle region inside the detector, which is consistent with the drift channel generated in the electric field distribution. A comparison of the electron concentration distribution of the P(r) and the second P(r) detectors shown in Figure 16a,b reveals that the second P(r) detector has a more uniform electron concentration distribution, indicating superior electrical performance of the detector.

## 6. Conclusions

In this paper, an innovative design of the silicon spiral drift detector (SDD) has been proposed. In our design method, the cathode gaps (g) under SiO_2_ are kept constant to minimize the surface leakage current. We allow the pitch profile $P\left(r\right)$ to vary in an arbitrary way to obtain the optimum electric field distribution. Exact solutions for the SDD with a spiral design $r=r\left(\theta \right)$ can be obtained for the hexagonal spiral SDD. According to the coordinates of each point of the spiral ring obtained by theoretical calculation, the SDD structure diagram can be drawn. Technology Computer-Aided Design (TCAD) is used to simulate the novel SDD in this work. We have systematically researched its respective electrical properties, such as electric field distribution, electric potential distribution, and electron concentration distribution. Simulation results have shown a uniform electric field and smooth electric potential and electron concentration profiles. It makes the SDD particularly outstanding in fields of space physics and photon science, such as pulsar X-ray detection and X-ray fluorescence spectrometers. This new design can be realized with the current SDD-processing technology, such as epitaxy, photolithography and ion implantation. In summary, our novel design of the silicon spiral drift detector can serve as a valuable method for the further improvement of SDD’s fields.

## Data Availability

The original contributions presented in the study are included in the article, further inquiries can be directed to the corresponding author.
